# Magnetic generation of normal pseudo-spin polarization in disordered graphene

**DOI:** 10.1038/s41598-021-94218-0

**Published:** 2021-07-22

**Authors:** R. Baghran, M. M. Tehranchi, A. Phirouznia

**Affiliations:** 1 Department of Physics, Shahid Beheshti University, 1983963113 G.C., Evin, Tehran, Iran; 2 Department of Physics, Azarbaijan Shahid Madani University, Tabriz, 53714-161 Iran; 3 Condensed Matter Computational Research Lab., Azarbaijan Shahid Madani University, Tabriz, 53714-161 Iran

**Keywords:** Physics, Condensed-matter physics, Spintronics

## Abstract

Spin to pseudo-spin conversion by which the non-equilibrium normal sublattice pseudo-spin polarization could be achieved by magnetic field has been proposed in graphene. Calculations have been performed within the Kubo approach for both pure and disordered graphene including vertex corrections of impurities. Results indicate that the normal magnetic field $B_z$ produces pseudo-spin polarization in graphene regardless of whether the contribution of vertex corrections has been taken into account or not. This is because of non-vanishing correlation between the $\sigma _z$ and $\tau _z$ provided by the co-existence of extrinsic Rashba and intrinsic spin–orbit interactions which combines normal spin and pseudo-spin. For the case of pure graphene, valley-symmetric spin to pseudo-spin response function is obtained. Meanwhile, by taking into account the vertex corrections of impurities the obtained response function is weakened by several orders of magnitude with non-identical contributions of different valleys. This valley-asymmetry originates from the inversion symmetry breaking generated by the scattering matrix. Finally, spin to pseudo-spin conversion in graphene could be realized as a practical technique for both generation and manipulation of normal sublattice pseudo-spin polarization by an accessible magnetic field in a easy way. This novel proposed effect not only offers the opportunity to selective manipulation of carrier densities on different sublattice but also could be employed in data transfer technology. The normal pseudo-spin polarization which manifests it self as electron population imbalance of different sublattices can be detected by optical spectroscopy measurements.

Benefits, like low-power operation and simple qubit-based spin language for data processing, storage and transfer technology applications, have made spintronics devices a reach field of research in recent years^1,2^. In fact, one of the central goals in spintronics is introducing an efficient mechanism to control both generation and detection of spin current/accumulation electrically^3,4^.

On the other hand, the conversion between different quantum numbers with different dephasing and diffusive lengths could be employed in the future data transfer technologies to obtain an optimized data transmission process. In this way, capability of charge-spin interconversion which arises from intrinsic spin–orbit interaction (SOI) has become one of the key phenomena in this field recently. This conversion has been realized by two mechanisms, spin Hall effect (SHE)^5–10^ and Edelstein effect (EE) some times known as inverse spin galvanic effect (ISGE)^11–16^.

The SHE and its Onsager reciprocal, inverse SHE known as effects in which interconversion between charge current and transverse electronic spin current takes place^17,18^. These effects have widely been studied in heavy metal layers^19–21^, semiconductors and two-dimensional materials^22^, spin-valve structures^23^ and superconductors^24^.The SHE has also been studied in disordered materials by taking into account vertex corrections within a diagrammatic based framework^25,26^ of perturbation theory. The SHE and inverse SHE, could be considered as two different efficient ways for generation and detection of pure spin current that carries a net angular momentum^4,8^.

The EE/ISGE and their Onsager reciprocals i.e. inverse Edelstein effect (IEE) and spin galvanic effect (SGE) were first propounded by Ivchenko and Pikus^27^, observed in *Te*^28^ and theoretically studied in two dimensional electron gas (2DEG) with broken inversion symmetry, in the presence of Dresselhaus and Rashba spin–orbit interactions (SOIs)^29–31^. The Rashba SOI results in interconversion between the charge current and non-equilibrium spin density. This correlation could be interpreted as spin-momentum locking which originates from the lack of inversion symmetry in low- dimensional systems such as semiconductors, and spin-momentum coupling in surface of topological insulators (TI)s^32–34^ and Weyl semi-metals^35^, oxide interfaces^16,35^ and two dimensional systems^36–41^.

Spin-momentum locking has also been realized in effective Hamiltonian of TIs. Akzyanov^42^ studied the spin conductivity of the surface states in a thin film of a TIs within the Kubo formalism where it has been shown that, these structures are promising materials for spintronics applications.

It must be highlighted that atomic texture of graphene-like systems, indicates an additional sublattice degrees of freedom called pseudo-spin, which could be related to a real angular momentum or other physically measurable effects^43,44^. This quantum degree of freedom has also been detected in several experiments directly^45–47^. The pseudo-spin concept is very substantial in Dirac systems due to the fact that many physical processes in these systems are completely pseudo-spin-dependent and could be understood just in this framework^48^.

Since the Dirac equation in graphene-like materials provides pseudo-spin-momentum coupling, it could be inferred that the pseudo-spin-momentum locking opens a very tempting way to realization of electric-based manipulation of pseudo-spin polarization for information and data processing applications. Several studies have been performed in the field of pseudo-spin manipulation in recent years^49–52^. Pesin and et al. show that charge current in single-layer graphene is accompanied with pseudo-spin currents. In 2014, Chen et al.^50^ investigated the possibility of extracting pseudo-spin polarization by means of electric field assisted electron emission.

Charge-spin interconversion which deals mainly with researches have been made in the field of SHE and EE covers a wide range of works from metal/oxide to Weyl semimetals and quantum wells^42,53–63^. So it could be deduced that unlike the spin and charge current inter-conversion which has been widely investigated, little attention has been paid to the fact that Dirac equation also provides a non-zero correlation between the spin of electron and sublattice pseudo-spin. Exploiting this linear coupling of spin and pseudo-spin provides both generation and manipulation of normal pseudo-spin polarization by magnetic field in Dirac systems.

Current paper presents spin to pseudo-spin conversion as an objective mechanism for generation and manipulation of non-equilibrium pseudo-spin polarization for both pure and disordered graphene within the Dirac point approximation. In the context of Kubo formalism and by taking into account the vertex corrections of impurities, it can be shown that the non-equilibrium spin polarization generates electron population imbalance on different sublattices i.e. pseudo-spin polarization. In addition, Onsager inverse of this effect, i.e. pseudo-spin to spin conversion, could be employed in detection of pseudo-spin density in Dirac materials with available magnetic measurement devices. Accordingly, the information which transfers with either of spin or pseudo-spin quantum numbers can be translated into each other. In addition, spin to pseudo-spin conversion provides a novel method to magnetic manipulation of electron populations on both A and B sublattices.

It has been shown that, spin to pseudo-spin response function in pure graphene is non-vanishing and valley-symmetric. Meanwhile, by taking into account the vertex corrections response function is weakened and the contribution of different valleys become non-identical. After all, the magnetic-induced pseudo-spin polarization as non-equilibrium charge population imbalance on different sublattices can be detected by the optical spectroscopy measurements.

## Methodology

The Hamiltonian of graphene as a Dirac-fermion structure with two *A* and *B* sublattices is generally described in the basis of $\{A\uparrow , A\downarrow , B\uparrow , B\downarrow \}$ as^64^

1 $$\begin{aligned} H^{\eta }_D = \hbar v_F\left( \eta k_x \tau _x+k_y \tau _y\right) +\eta \lambda _{so} \tau _z \otimes \sigma _z + \lambda _{R1} \left( \eta \tau _x \otimes \sigma _y- \tau _y \otimes \sigma _x \right) . \end{aligned}$$

In which, $\hbar v_F=\frac{\sqrt{3}}{2}at$ where *a* is lattice constant and *t* is the first nearest neighbors hopping energy, $\tau = (\tau _x, \tau _y, \tau _z)$ refers to sublattice degree of freedom called pseudo-spin which could be represented by well-known Pauli matrices similar to spin operators $\sigma _\alpha =(\sigma _x,\sigma _y,\sigma _z)$. Where, $\sigma _\alpha$ being the $\alpha$-component of the electron spin.

In-plane components of pseudo-spin operator $\tau _{x},~\tau _{y}$ represent nearest neighbors electron hopping between *A* and *B* sublattices, meanwhile, the out of plane operator $\tau _z$ indicates sublattice quantum number *A* or *B*. Each of these sublattices can be described by its own Bloch wave function denoted by $\psi _{A}$ and $\psi _{B}$. $\psi _{A/B}$ remains invariant under the action of $\tau _z$ meanwhile, in plane pseudo-spin operators $\tau _{x(y)}$ changes the state of electrons wave function from $\psi _A$ to $\psi _B$ and vice versa. $\eta$ is the valley index that gets $+1$, $-1$ values for the *K* and $K'$ points respectively. $\lambda _{so}$ indicates the strength of intrinsic SOI and finally the strength of externally induced inversion asymmetry that leads to extrinsic Rashba SOI is identified by $\lambda _{R1}$.

Remarkably, the intrinsic SOI is of key importance in spin to pseudo-spin response function. However, it can be shown that for low energy graphene ok, nonzero response function of spin to pseudo-spin conversion crucially depends on the presence of extrinsic Rashba coupling. As The numerical results indicate, the extrinsic Rashba interaction plays an important role in effectiveness of the intrinsic SOI in this process. At zero Rashba coupling strength ($\lambda _{R1}\rightarrow 0$) and close to the Dirac points, low energy Hamiltonian of graphene is reduced to $H^{\eta }_D \simeq \hbar v_F\left( \eta k_x \tau _x+k_y \tau _y\right) +\eta \lambda _{so}\tau _z \otimes \sigma _z$ that commutes with spin operator.

### Response functions based on bare and dressed Green’s functions

It can be realized that the charge population imbalance can be generated by non-equilibrium spin polarization. This spin polarization can be provided by a time-dependent Zeeman interaction $H_{Z}(t) = \mu _B \sigma _zB_z(t)$ as a physical perturbation where the normal magnetic field $B_{z}(t)$ varies harmonically with time. Referring to Eq. (1) it could be inferred that low energy effective Dirac Hamiltonian of graphene can provide spin to pseudo-spin conversion. As the intrinsic SOI guarantees the correlation between the spin of electron and sublattice pseudo-spin. Accordingly, it could superficially be interpreted that the intrinsic SOI provides spin to pseudo-spin conversion. Nevertheless, it can be shown that for low energy graphene, the non-vanishing spin to pseudo-spin conversion crucially depends on the presence of extrinsic Rashba coupling.

Spin to pseudo-spin conversion for a pure system could be realized as DC limit of $\tau _z$ response to the Zeeman interaction that given by the following expression within the Kubo approach^65^,

2 $$\begin{aligned} \chi ^{(0)}_{\sigma _z\tau _z } =\lim _{\omega \rightarrow 0}\sum _{\alpha , \alpha '}\frac{<\alpha |\tau _z|\alpha '><\alpha '|\sigma _z|\alpha >}{E_{\alpha }-E_{\alpha '}+\hbar \omega + i \varepsilon } \end{aligned}$$

where $|\alpha>$ and $E_{\alpha }$ are the unperturbed eigenstates and eigenvalues respectively and $\varepsilon$ is a small positive. According to the above expression, it can easily be realized that when the motive perturbation $\sigma _{z}$ commutes with total Hamiltonian, the response function ($\chi ^{(0)}_{\sigma _z \tau _z}$) identically vanishes. Equivalently, in the absence of extrinsic Rashba interaction, low energy Hamiltonian of graphene is reduced to $H^{\eta }_D \simeq \hbar v_F\left( \eta k_x \tau _x+k_y \tau _y\right) +\eta \lambda _{SO}\tau _z \otimes \sigma _z$ which commutes with $\sigma _{z}$. Meanwhile, in the presence of extrinsic Rashba coupling motive force that associated with $\sigma _{z}$ operator does not commute with Dirac Hamiltonian which accordingly results in non-vanishing spin to pseudo-spin response function. This fact can easily be understood if we consider that the perturbations commuting with the Hamiltonian cannot disturb its eigenstates and generate a change in the mean value of the observables i.e. a response in the system. Finally, this would suggest that the extrinsic Rashba SOI possess a fundamental impact on effectiveness of the intrinsic SOI in spin to pseudo-spin conversion.

In the current study, it should be noted that we have demonstrated how non-equilibrium pseudo-spin polarization could be produced by normal magnetic filed by exploiting of non-vanishing correlation between the spin of electrons and pseudo-spin texture in graphene.

**Figure 1:**
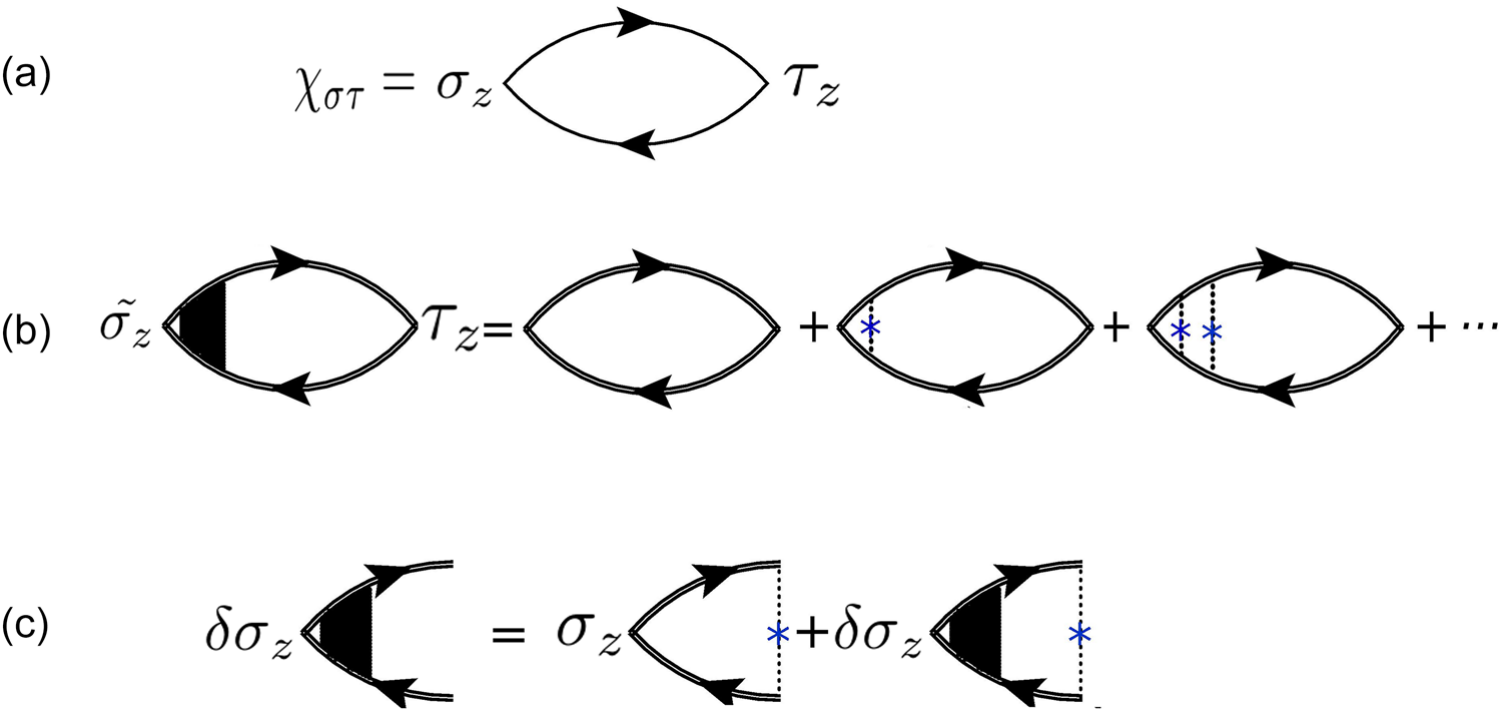
Feynman bubble diagrams; the graphical representation of spin to pseudo-spin response function. Single lines are undressed Green’s functions of the sample while double lines denote the dressed Green’s functions. Cross shape blue points refer to impurity and dashed lines show scattering from impurity potential^26^. (**a**) Unperturbed response function of the spin and pseudo-spin. (**b**) Ladder diagrams correspond to the corrections of the response function which come from successive interactions of electron-hole pair with impurities that leads to normalized vertex spin operator. (**c**) The diagrammatic approach of electron-hole pair interactions with impurities results in iterative equation of vertex correction.

The influence of impurities can be considered at different levels i.e. up to the Born approximation that modifies the bare retarded and advanced Green’s function or by normalizing the response function via the vertex corrections. The spin to pseudo-spin conversion can be expressed by bare Kubo^26,30,66^ response function, $\chi ^{\eta (0)}_{\sigma _z\tau _z}$, as shown in Fig. 1a

3 $$\begin{aligned} \chi ^{\eta (0)}_{\sigma _z\tau _z}= \int \frac{d^2k}{(2\pi )^2} Tr[\tau _z G^{R(\eta )}_{0}(E_F,k) \sigma _z G^{A(\eta )}_{0}(E_F,k)]. \end{aligned}$$

where $\tau _z$ and $\sigma _z$ operators could be written as $\tau _z = \tau _z \otimes I_{\sigma }$ and $\sigma _z = I_{\tau } \otimes \sigma _z$ respectively in pseudo-spin, spin Hilbert space and $G^{A/R}_{0}$ denote the advanced/retarded un-dressed Green’s functions in the absence of disorders that given by,

4 $$\begin{aligned} G^{A/R(\eta )}_{0}(E,k)=[E\times I- {\hat{H}}^{\eta }_D {\mp } i0^{+} ]^{-1}, \end{aligned}$$

where *I* is $4\times 4$ identity matrix. In the presence of impurities with the typical potential $V_{im}$, a level broadening is introduced by the imaginary part of the self-energy, $\Sigma$.

Dyson equation that describes Green’s function in term of disorder averaged self- energies reads

5 $$\begin{aligned} \Sigma ^{A/R}& = {} <V_{im}+V_{im}G^{A/R}_{0}V_{im}>_{dis} \end{aligned}$$

6 $$\begin{aligned} {\hat{H}}& = {} {\hat{H}}^{\eta }_D +\Sigma \end{aligned}$$

7 $$\begin{aligned} G& = {} G_{0}+G_{0}\Sigma G_{0}+G_{0}\Sigma G_{0}\Sigma +...\nonumber \\& = {} G_{0}+G_{0}\Sigma G. \end{aligned}$$

where $<>_{dis}$ in Eq. (5) refers to disorder configuration average. Real part of the self-energy introduces an energy shift which could be ignored by redefinition of eigen-energies and the imaginary part of the self-energies within the Born approximation reads $Im \Sigma ^{A/R}={\mp } i\hbar /2\tau _{im}$. Here, $\tau _{im}$ is the relaxation time which directly relates to level broadening concept. Then the advanced/retarded dressed (effective) Green’s functions could be written as

8 $$\begin{aligned} G^{A/R(\eta )}(E,k)=[E\times I-{\hat{H}}^{\eta }_D {\mp } \frac{i\hbar }{2\tau _{im}} ]^{-1}, \end{aligned}$$

In which $G^{A/R}$ indicates dressed (effective) Green’s function.

The impurity potential $V_{im}$ could be written as^26^

9 $$\begin{aligned} V_{im} = u_{0}R^{2} \displaystyle \sum _{j} M_{4\times 4} \delta (\mathbf {r}-\mathbf {r_{j}}). \end{aligned}$$

In which summation runs over the position of impurities, ${\mathbf {r}}_j$, $u_0$ is the power of impurity potential, *R* is length scale refers to impurity potential range and $M_{4\times 4}$ is the scattering matrix that can be presented by

10 $$\begin{aligned} M = M_0 + M_{SO}= \tau _0 \otimes \sigma _0 + \tau _{z} \otimes \sigma _{z}. \end{aligned}$$

where $M_0$ denotes the scalar point-like scatterers and $M_{SO}$ refers to spin–orbit type impurities. Accordingly, the intrinsic SOI of both host and disorder atoms have the same functional form in pseudo-spin, spin four-band space. Actually, each elements of the scattering matrix, *M*, refers to a transition that could be made by impurities in $|\tau _z>\otimes |\sigma _z>$ space. As mentioned, real part of the self-energy just leads to an energy shift which could be ignored by redefinition of eigen-energies, meanwhile the imaginary part of the self-energies within the Born approximation is given by $Im \Sigma ^{A/R}={\mp } i\hbar /2\tau _{im}$. The scattering time within the first Born approximation is given by

11 $$\begin{aligned} \frac{1}{\tau ^{\lambda }_{im}(k)} = \frac{2\pi }{\hbar }\sum _{{\lambda '}{k'}} |<k\lambda |V_{im}|{k'} {\lambda '}>|^2 \delta (E_{k\lambda }-E_{k' \lambda '}) \end{aligned}$$

where $\lambda (\lambda ')$ indicates band index and $|k \lambda >$ is eigenstate of Dirac Hamiltonian, Eq. (1) . Elastic scatterings are identified by the Dirac delta function in Eq. (11). This means that elastic scatterings which have been taken into account in the dressed Green’s functions can contribute to relaxation of polarizations due to the conductor phase of system. Meanwhile, they cannot contribute to the inter-band transitions of insulating phase.

The generalized dressed Kubo response function, $\chi ^{(\eta )}_{\sigma _z\tau _z}$ could be written as

12 $$\begin{aligned} \chi ^{(\eta )}_{\sigma _z\tau _z}= \int \frac{d^2k}{(2\pi )^2} Tr[\tau _zG^{R(\eta )}(E_F,k) \sigma _z G^{A(\eta )}(E_F,k)] \end{aligned}$$

### Vertex corrections

The influence of impurities could be effectively formulated by considering different ways of interaction. First, independent interactions of electrons and holes with impurities. This effect can generally be captured by scattering time that could be obtained within the Born approximation. The scattering time effectively captures the influence of independent scatterings in the self-energies. Accordingly, bare Green’s functions are replaced with dressed ones. On the other hand vertex corrections normalizes spin to pseudo-spin response function by taking into account the correlated interaction of electron-hole pair with a single impurity. This type of process appears as a set of pair interactions which could be shown as ladder type couplings depicted in Figs 1b,c. By means of vertex corrections as a diagrammatic based concept the influence of correlated interactions can be included.

As shown in Fig. 1, vertex corrections indicates the electron and hole propagators correlation via impurity mediated interactions. Actually vertex correction can be considered as a parallel set of independent scatterings, so in the limit of ladder approximation, it leads to well-known Bethe–Salpeter self-consistence equations as^26^

13 $$\begin{aligned} \delta \sigma _z& = {} \bar{\sigma _z}+ n \displaystyle \sum _{k} V_{im}G^{R}_{k} \delta \sigma _z G^{A}_{k}V_{im}, \end{aligned}$$

14 $$\begin{aligned} \bar{\sigma _z}& = {} n \displaystyle \sum _{k} V_{im}G^{R}_{k} \sigma _z G^{A}_{k}V_{im}. \end{aligned}$$

where *n* denotes the impurity density. Using the dressed Green’s functions the vertex corrections of normal spin, $\delta \sigma _z$, can be obtained by the following iterative equation

15 $$\begin{aligned} \delta \sigma _z& = {} n \displaystyle \sum _{k} V_{im}G^{R}_{k}(\sigma _z+\delta \sigma _z)G^{A}_{k}V_{im}\nonumber \\& = {} \frac{n}{(2\pi )^2}\int d^2k V_{im} G^{R}_{k}(\sigma _z+\delta \sigma _z)G^{A}_{k}V_{im}. \end{aligned}$$

This iterative equation can be solved for weak or low density impurities numerically. Finally, after substitution of $\sigma _z$ with $\sigma _z+\delta \sigma _z$, in Eq. (12) one can write the final expression for dressed response function under vertex corrections as (computational codes have been provided in supplementary material)

16 $$\begin{aligned} \chi _{\sigma _z\tau _z}= \int \frac{d^2k}{(2\pi )^2} Tr[\tau _z G^{R}_{k}(\sigma _z+\delta \sigma _z)G^{A}_{k}]. \end{aligned}$$

## Results and discussions

Here, we present the numerical results which mainly contain $\chi ^{\eta }_{\sigma _z\tau _z}$ response function in terms of the extrinsic Rashba coupling strength ($\lambda _{R1}$) for both pure and disordered graphene in it’s metallic phase. All calculations have been performed for $\hbar v_F = 5.96(eV.A^{0})$, $\lambda _{SO}= 1.3(\mu eV)$ and $K_BT=0$. The results have been obtained through the numerical computations based on theoretical approach have been explained in previous sections.

**Figure 2:**
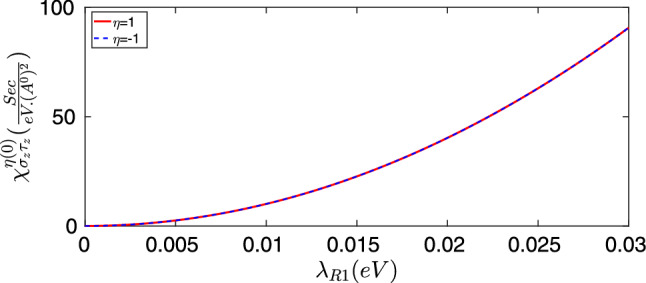
Spin to pseudo-spin bare response function of pure graphene in terms of the extrinsic Rashba coupling strength for $E_{F}=0.08(eV)$.

Figure 2 represents the behavior of spin to pseudo-spin response functions of both valleys $\chi _{z}^{\eta }{_{\tau _z}}$ in terms of the extrinsic Rashba coefficient $\lambda _{R1}$. As to Fig. 2 it can be deduced that in the absence of disorders spin to pseudo-spin conversion and it’s relevant response function does not vanish. This is due to the fact that the co-existence of extrinsic Rashba and intrinsic SOIs guaranties the non-vanishing spin, pseudo-spin correlation that could be achieved even in the absence of disorders. The obtained response function vanishes at the limit of $\lambda _{R1}\rightarrow 0$ and increases with increasing $\lambda _{R1}$. Accordingly, the Rashba interaction can provide a framework for effective contribution of intrinsic SOI on spin to pseudo-spin response function. In this way, decreasing the Rashba coupling strength suppresses the contribution of intrinsic SOI and at the limit of $\lambda _{R1}\rightarrow 0$ where the spin to pseudo-spin response function vanishes. Hence, the intrinsic SOI has not an independent contribution in spin to pseudo-spin conversion. According to Fig. 2 it could be demonstrated that, for the case of pure graphene spin to pseudo-spin response function is valley-symmetric and both *K* and $K^{\prime }$ valleys contribute identically to spin to pseudo-spin conversion.

Although the non-zero spin to pseudo-spin response function could be obtained even in the absence of disorders we tend to expand our calculations to realistic disordered graphene where the electron-impurity scattering has been taken into account. For the case of elastic relaxations within the Born approximation, the response function for both different valleys goes to zero. This is due to the fact that scalar scattering matrix effectively suppresses the relevant response functions of both valleys in metallic phase of graphene.

**Figure 3:**
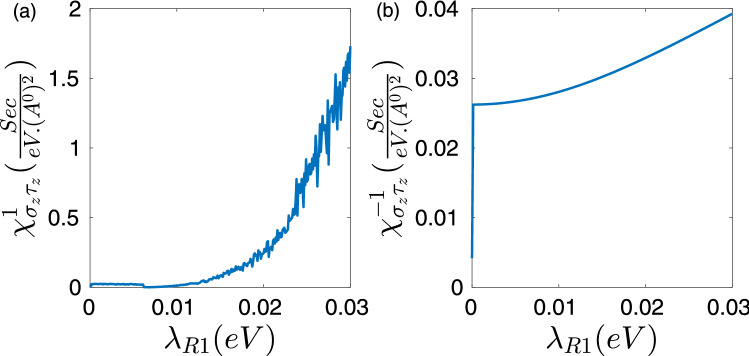
Spin to pseudo-spin response function of disordered graphene at *K* (**a**) and $K^{\prime }$ (**b**) Dirac points in terms of the extrinsic Rashba coupling strength by taking into account the vertex corrections for $E_F=0.08(eV)$ .

Figure 3 shows the behavior of spin to pseudo-spin response functions $\chi ^{\eta }_{\sigma _z\tau _z}$ in terms of the extrinsic Rashba SOI strength $\lambda _{R1}$ for disordered graphene in the presence of vertex corrections for $E_F=0.08(eV)$. According to Fig. 3, it can be inferred that vertex corrections considerably weakens the obtained response functions of both valleys. Referring to Eq. (1) it could be realized that very close to the Dirac points i.e. at the limit of $k\rightarrow 0$ the Dirac point Hamiltonian of graphene can be written approximately as $H^\eta _D \approx \lambda _{R1}(\eta \tau _x \otimes \sigma _y-\tau _y \otimes \sigma _x)$ since the intrinsic SOI is very small. The scalar part of relaxations $<k^{\prime }\lambda ^{\prime }|M_0|k\lambda>$ refers to intra-band transitions while the spin–orbit type relaxations $<k^{\prime }\lambda ^{\prime }|M_{SO}|k\lambda>$ determine the inter-band transitions. In this way, both independent and correlated scatterings can effectively contribute to relaxation process of motive $\sigma _z$ operator via both inter-band and intra-band transition.

Vertex corrections of impurities normalizes the spin to pseudo-spin response function by several orders of magnitude for both valleys. This is due to the fact that, the band energies of the Dirac point Hamiltonian and valley points are not pseudo-spin resolved and spin-polarized respectively in graphene. Accordingly both type of the inter-band and intra-band scatterings can effectively contribute in the relaxation of motive $\sigma _z$-polarization by a series of interactions that mediated by impurities between electrons and holes.

Moreover, numerical results indicate that by taking into account the vertex corrections spin to pseudo-spin conversion is also accompanied with generation of non-equilibrium valley polarization. In other words, in this case different valleys contribute non-identically to the response function.

So a small valley polarization could be observed which has been defined as

17 $$\begin{aligned} P=\frac{\chi ^{1}_{\sigma _{z}\tau _{z}}-\chi ^{-1}_{\sigma _{z}\tau _{z}}}{\chi ^{1}_{\sigma _{z}\tau _{z}}+\chi ^{-1}_{\sigma _{z}\tau _{z}}} \end{aligned}$$

This valley-asymmetry that appears in the presence of vertex corrections could be explained by the fact that spin–orbit type scattering matrix breaks the inversion symmetry. It could be understood by the fact that inversion operator (*I*) changes the A-sublattice to B-sublattice and vice versa. This means that it changes the sign of $\tau _z$. In other words, inversion operator in graphene-like structures can be given by x-component of the sublattice pseudo-spin operator ($\tau _x$). As $I^{-1}\tau _z I = \tau _x \tau _z \tau _x = -\tau _z$, it could be inferred that unlike the spin, $\tau _z$ can be considered as polar component^67^. Accordingly, we have $I^{-1}M_{SO}I = \tau _x \tau _z\sigma _z \tau _x = -\tau _z\sigma _z= -M_{SO}$ which means that the spin–orbital component of scattering matrix breaks the inversion symmetry and therefor gives rise to non-identical contributions of different valleys.

As depicted in Fig. 2, for the case of pure graphene, the obtained response functions are increased by several orders of magnitude and show monotonic behavior as a function of $\lambda _{R1}$ with identical contributions of different valleys.

It must be commented that the origin of the oscillations in Fig. 3a are not completely understood, and they could be the result of numerical instabilities in the self-consistence calculations used in Eqs. (15 and 16).

It must be noted that calculations performed in this work are valid at weak impurity limit in which the numerical iterations of the vertex corrections can be converged. In this way, both density and strength of the impurities are limited to a convergence range of the Bethe–Salpeter equation Eq. (15).

Hence, due to non-vanishing correlation between the spin of electron and sublattice pseudo-spin that has been guaranteed by the co-existence of extrinsic Rashba and intrinsic SOIs the normal magnetic field $B_z$ breaks the inversion symmetry by inducing a population imbalance between the *A* and *B* sublattices. So it can open up a band-gap in graphene structure which determines a cutoff frequency in optical absorption spectra. Therefore, if population imbalance could be established between the *A* and *B* sublattices the inversion symmetry of effective electron-electron interaction is broken, which provides a measurable optical effects. Band gap of the graphene can effectively be measured by optical methods^68^. In other words, spin to pseudo-spin conversion could be realized as an easy way to selective manipulation of electrons population in either of *A* and *B* sublattices by an accessible normal magnetic field. On the other hand, charge population imbalance could result in non-equilibrium spin polarizations via the Onsager reciprocity relation which can be used in data transfer technologies. In summary, spin to pseudo-spin conversion could be considered as a practical way through magnetic generation and manipulation of non-equilibrium normal sublattice pseudo-spin polarization in graphene that could be employed in data transfer technology.

## Concluding remarks

A novel type of conversion which could be called spin to pseudo-spin conversion has been proposed for both pure and disordered graphene. This conversion originally arises from the extrinsic Rashba and intrinsic spin–orbit interactions. In this way, the spin to pseudo-spin response functions, $\chi ^{\eta }_{\sigma _z\tau _z}$, have been computed in the context of Kubo formalism and by taking into account the contribution of vertex corrections. For pure graphene, the response function increases by increasing the extrinsic Rashba coupling strength ($\lambda _{R1}$) with identical contributions of different valleys. Meanwhile, in the presence of vertex corrections of impurities the spin to pseudo-spin response function is suppressed while two different valleys contributions are non-identical due to inversion symmetry breaking that caused by the spin–orbital part of scattering matrix.

The magnetically induced non-equilibrium charge distribution gives rise to sublattice inversion symmetry breaking of effective electron-electron interaction field that can be appeared as a change of cutoff energy in optical absorption spectra in graphene. Therefore, spin to pseudo-spin conversion could be measured by the well-known optical spectroscopy measurements.

## Supplementary Information

Supplementary Information 1.

Supplementary Information 2.

Supplementary Information 3.

Supplementary Information 4.

Supplementary Information 5.

Supplementary Information 6.

Supplementary Information 7.

Supplementary Information 8.

Supplementary Information 9.

Supplementary Information 10.
